# Artificial intelligence and patient reported outcomes in ophthalmology

**DOI:** 10.1186/s41687-026-01033-0

**Published:** 2026-03-07

**Authors:** Luke Tran, Himal Kandel, Shayan Lahijanian, Stephanie L. Watson

**Affiliations:** The University of Sydney, Sydney, Australia

**Keywords:** Artificial intelligence, Ophthalmology, Patient reported outcomes, Review

## Abstract

**Purpose:**

Artificial intelligence (AI) has the potential to revolutionise the delivery of ophthalmic healthcare worldwide. Whether AI is making a meaningful difference or is acceptable for patients, however, remains unclear. Patient reported outcomes (PROs) allow researchers to answer these questions and smooth the path to clinical deployment. This review aims to investigate how PROs are being applied to the development and evaluation of ophthalmic AI technology and explore any underlying reasons why PROs may be currently underutilised.

**Method:**

A systematic search of electronic databases for studies and clinical trials applying PROs in the development and evaluation of ophthalmic AI was conducted from date of inception to February 2025.

**Results:**

Fifty articles applied a PRO to ophthalmic AI, from which 14 interventional studies and 24 unique validated PROs were identified. There was a rapid year-on-year increase in PRO utilisation beginning in 2020 until 2024. PROs were concentrated in economically advanced countries, were generic (58%) rather than disease-specific (40%), and most often were used as evaluator metrics (50%), or input (44%) for the AI model. Few articles investigated consumer-ready technologies (12%).

**Conclusion:**

Low research priority, the nascent state of AI in ophthalmology, and lack of high quality accumulated PROs data were identified as possible barriers to realising the full potential of PROs in ophthalmic AI. Investment into the development of robust validated PROs and the inclusion of PROs in routine data collection may catalyse the development of AI technologies capable of making the greatest meaningful difference to patients’ quality of life.

**Supplementary Information:**

The online version contains supplementary material available at 10.1186/s41687-026-01033-0.

## Introduction

Artificial intelligence (AI) has shown great potential as a means to streamline healthcare delivery and improve the capacity of health systems worldwide [1, 2]. Ophthalmology stands to benefit greatly from advances in AI technology with promising results achieved in the workup and management of various leading causes of blindness including diabetic retinopathy, macular degeneration, and glaucoma [3–5]. As research continues to progress, concerns have been raised regarding the realities of clinical implementation for these AI tools and more specifically, whether these AI technologies are acceptable and make a meaningful difference from the patient perspective [6, 7].

Patient reported outcomes (PROs) are reports of a patient’s health status or their experience with healthcare unfiltered by any external entity. PROs may take many forms including validated patient reported outcome measures (PROMs) which measure health status, function, and impacts of treatment or illness, patient reported experience measures (PREMs) which gauge patient perspectives, experience and satisfaction, social media posts, usability measures, and various non-validated questionnaires and surveys [8–12]. PROs provide researchers with a wealth of patient-centric information and when applied to the development and evaluation of ophthalmic AI tools, PROs allow researchers to effectively address these concerns about meaningful differences and acceptability of the AI model for patients and smooth the path to clinical deployment [13].

Despite these purported benefits, a recent review of the role of PROs in clinical trials of AI health technology found that the incorporation of PROs in ophthalmic trials was significantly lacking compared to other clinical areas but did not provide any explanation to explain the discrepancy [14]. To that effect, this review aims to provide a comprehensive overview of how PROs are being applied to ophthalmic AI technology and explore any underlying reasons why PROs may be currently underutilised.

## Methods

A systematic search of electronic databases including PubMed, Embase, Medline, and ClinicalTrials.gov was conducted from date of inception till February, 2025 for papers and trials applying a PRO to ophthalmology relevant AI models and algorithms. The full search strategy can be found within supplementary material. Articles were subsequently exported into Covidence [15] where exact duplicates were automatically filtered out. Two authors (LT, SL) then independently screened abstracts to find relevant articles. Studies meeting the established eligibility criteria subsequently underwent full text evaluation for inclusion into the systematic review. Discrepancies in screening between the first two authors were deliberated to reach consensus. Instances where disagreements remained were brought to a third author for a final decision (HK).

### Eligibility criteria

All studies investigating an ophthalmology relevant AI model and that employed at least one PRO were included. Papers investigating systemic diseases with ophthalmic manifestations were also accepted provided the data on ocular symptoms were incorporated into the AI model. All forms of AI from traditional machine learning to deep learning algorithms were included. Furthermore, the definition of a PRO was kept broad to provide a more comprehensive measure of the value of the patient perspective in the field of ophthalmology as follows [12]. For this review, all sources of patient perspectives in the form of PROMs, PREMs, usability measures, questionnaires, surveys, forums or social media were considered a form of PRO regardless of whether or not they were associated with a formal validation study.

Studies where the PRO and AI did not interface were excluded. Where multiple studies reported on AI models developed on the same dataset, only the most recent study was included. No limits were set regarding country of origin and age of participants. Papers investigating non-human subjects and papers not available in English were excluded. Given that research within the field of computer science is often disseminated via conference presentations, relevant conference abstracts were included except in cases where there was insufficient data to populate the data extraction table. Reviews, protocols, letters, editorials and case reports were excluded.

### Data extraction and analysis

Data from the included studies were extracted via a standardised data extraction table. The following data was extracted from each article: title; authors; date of publication; country of AI deployment; disease of interest; subspecialty; form of AI; role of AI; type of algorithm; AI user; type of PRO; generic vs. disease-specific PRO; PRO endpoint positioning; PRO as input, output or evaluator metric; and validation status. Descriptive statistics and illustrative figures were formulated in Microsoft Excel (version 2108) [16].

## Results

**Fig. 1:**
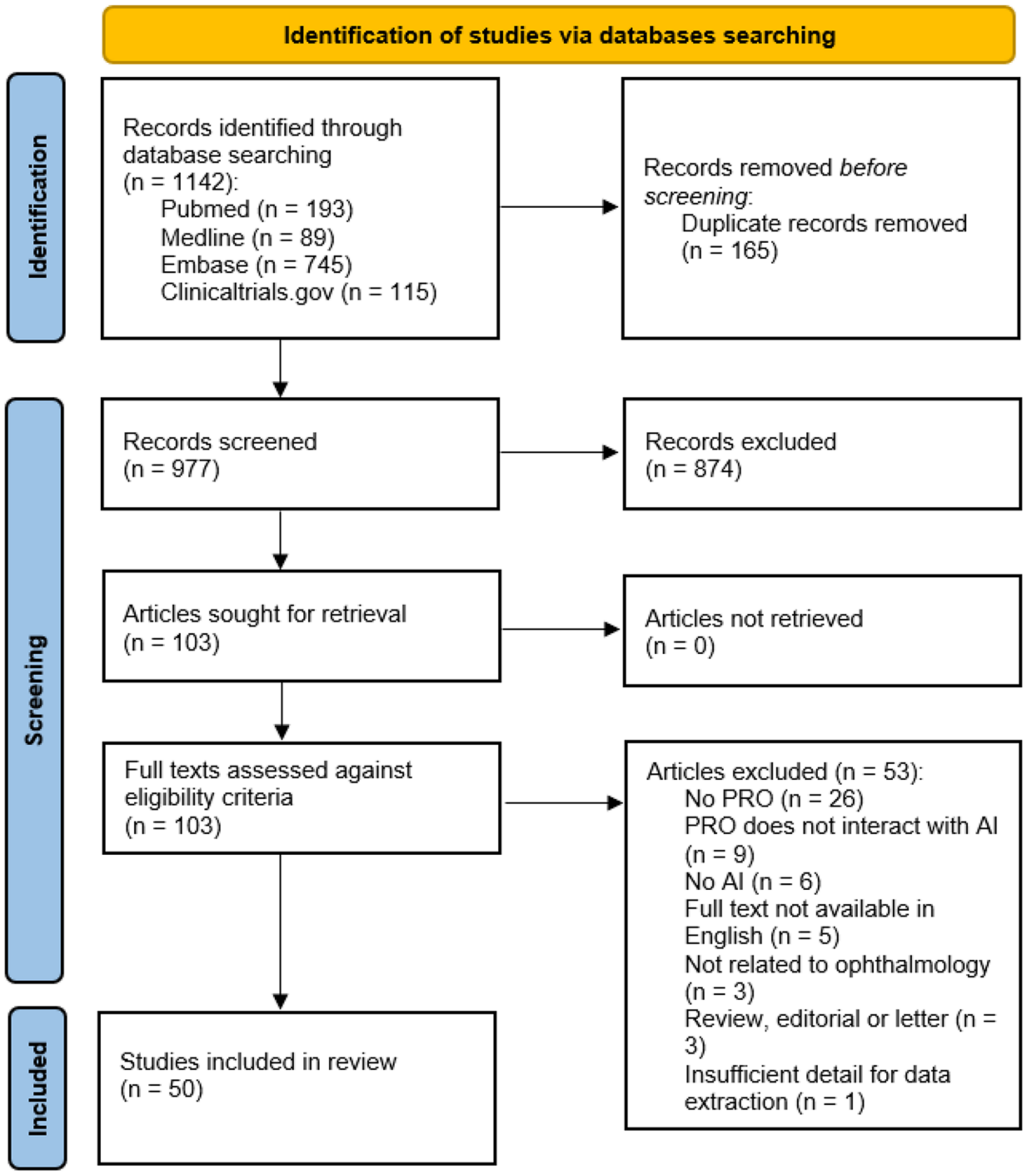
Flow diagram of studies identified by database search

The search retrieved 1142 articles of which 165 were automatically marked as exact duplicates, leaving 977 articles for screening. Screening of titles and abstracts yielded 103 articles for full text screening. Application of the eligibility criteria to the remaining 103 records resulted in 50 articles suitable for inclusion in the review. A graphical depiction of the study selection process is viewable in Fig. 1 and a summary table of key PRO results from peer-reviewed research papers can be found in Supplementary Tables 1–3.

### Articles by publication type, year and country of origin

Of the 50 studies included in the review, 26 (52%) were published peer-reviewed clinical research papers [17–42], there were 9 (18%) conference abstracts [43–51], and 15 (30%) clinical trials (Supplementary Material). Regarding study design, most articles were observational in nature (*n* = 36, 72%) with fewer studies directly testing an intervention (*n* = 14, 28%).

**Fig. 2:**
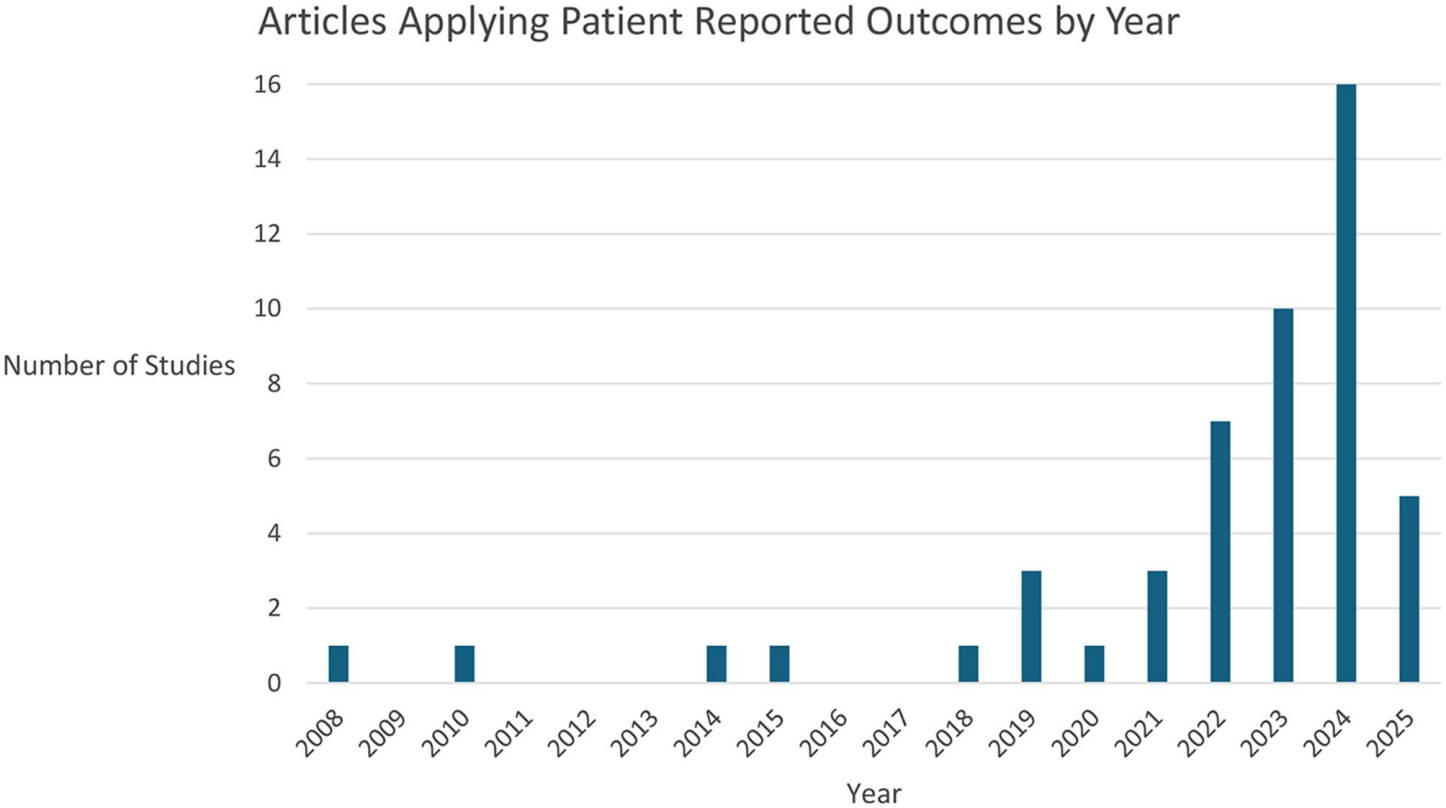
Articles included in the systematic review of patient reported outcomes and ophthalmic artificial intelligence stratified by year of publication

As depicted in Fig. 2, the number of articles applying a PRO to an ophthalmology-relevant AI technology increased substantially from 1 article in 2020 to a high of 16 articles in 2024. Prior to 2020, the inclusion of PROs was sporadic. As of February 2025, there were already 5 articles incorporating at least one PRO in the evaluation and development of ophthalmic AI technology.

**Fig. 3:**
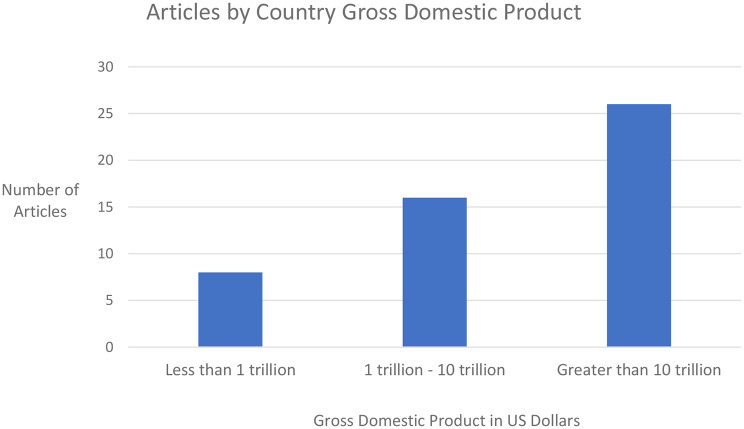
Articles included in the systematic review of patient reported outcomes and ophthalmic artificial intelligence stratified by country gross domestic product

Figure 3 demonstrates the distribution of PRO applying articles in ophthalmic AI as a function of country gross domestic product (GDP) [52]. Countries with a GDP less than 1 trillion US dollars produced the fewest articles (*n* = 8, 16%), followed by countries with a GDP between 1 and 10 trillion dollars (*n* = 16, 32%), and countries with a GDP over 10 trillion dollars produced the greatest number of articles (*n* = 26, 52%).

**Fig. 4:**
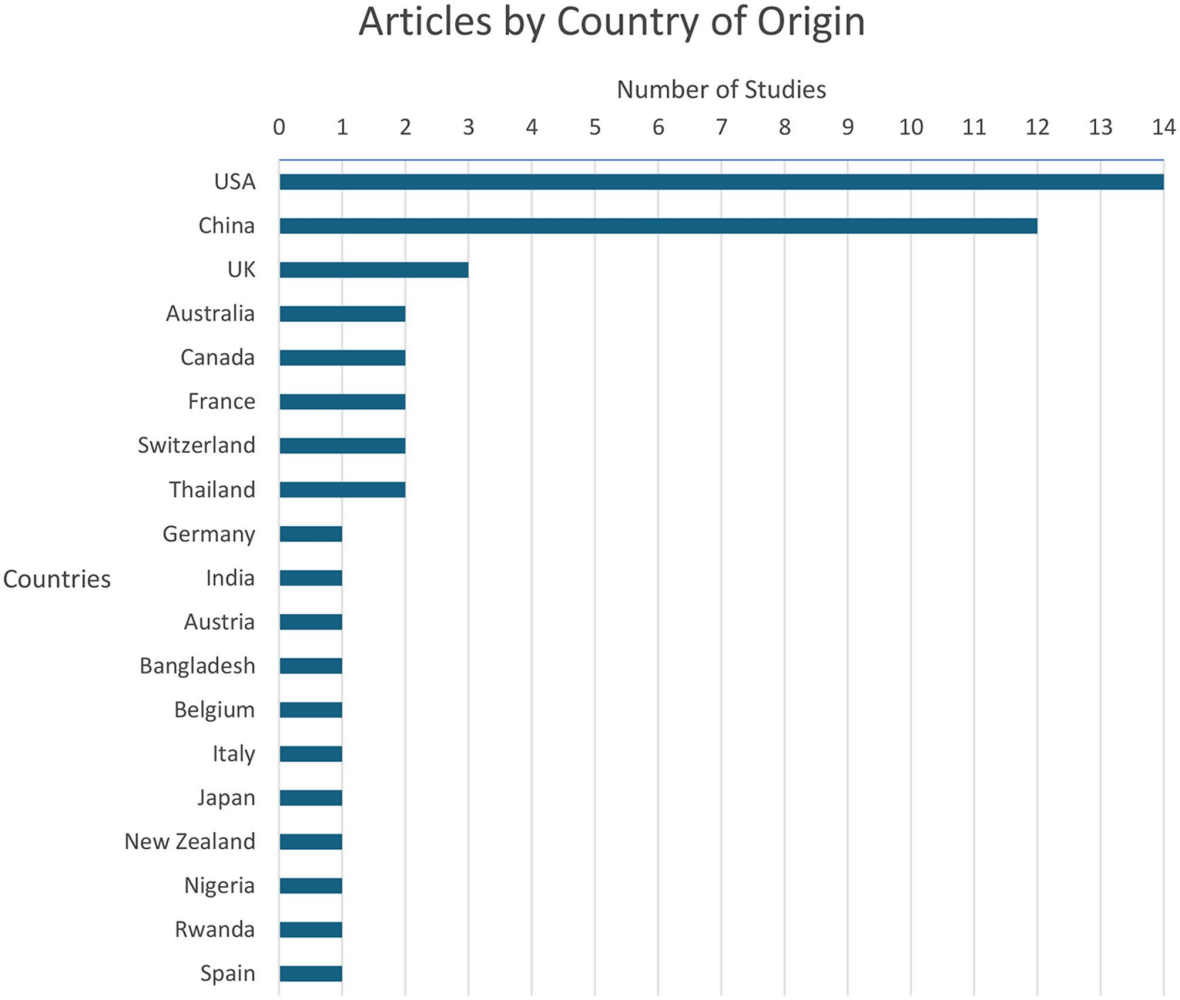
Articles included in the systematic review of patient reported outcomes and ophthalmic artificial intelligence stratified by country of origin

The USA had the largest number of articles (*n* = 14, 28%) employing PROs to ophthalmic AI followed by China with 12 (24%) articles and the UK with 3 (6%) articles. The remaining 21 articles were distributed between 16 countries with one or two articles per country (Fig. 4).

### Ophthalmic subspecialty

**Fig. 5:**
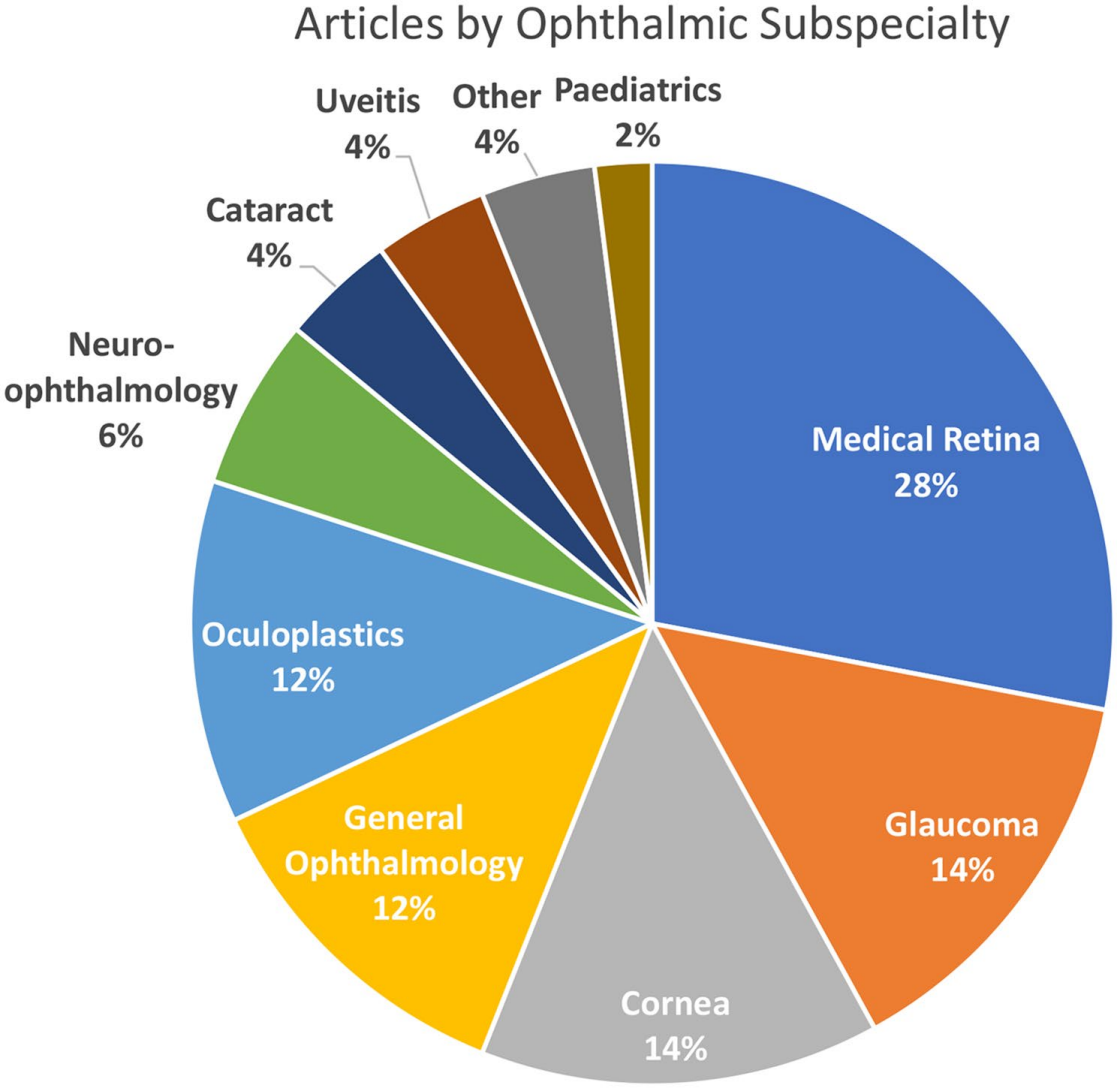
Articles included in the systematic review of patient reported outcomes and ophthalmic artificial intelligence stratified by ophthalmic subspecialty

The 50 included articles were also analysed by ophthalmic subspecialty (Fig. 5). Medical retina comprised the bulk of articles (*n* = 14, 28%), followed by glaucoma (*n* = 8, 14%), cornea (*n* = 7, 14%), general ophthalmology (*n* = 6, 12%), oculoplastics (*n* = 6, 12%), and neuro-ophthalmology (*n* = 3, 6%). The remaining 7 articles were distributed between cataract, uveitis, other, and paediatrics, each with 4% of the articles or less. Notable subspecialty omissions included surgical retina, ocular oncology and strabismus which did not return any articles employing PROs on ophthalmic AI technology in this search.

### PROM characteristics

Table 1 provides an overview of the PRO characteristics found in this review. Within the included studies four broad categories of PROs were identified. PROMs were most commonly utilised in the studies (*n* = 23, 44.2%). PREMs were also found in 19 studies (36.5%). Social media as a form of PRO was encountered in 7 (13.5%) articles, and usability measures were least reported and found in only 3 (5.8%) articles.

Patient reported outcomes identified tended to be generic (*n* = 29, 58%) rather than disease-specific (*n* = 21, 40%) with only 1 study making use of both a generic and disease-specific PRO (2%). Moreover, PROs were primarily used as an evaluator metric for the AI model (*n* = 25, 50%) or as an input into the model (*n* = 23, 46%). Few articles employed PROs as both an input and output to the AI technology (*n* = 3, 6%) and no articles used PROs solely as an algorithm output.

There was a balanced distribution found in articles employing validated PROs (*n* = 25, 50%) against non-validated PROs (*n* = 25, 50%). From the 25 articles employing validated PROs, there were 24 unique tools identified with the most common being the National Eye Institute Visual Function Questionnaire-25 (*n* = 5, 20%), the EQ-5D (*n* = 3, 12%), and the Graves’ Ophthalmopathy-Quality of Life Questionnaire (*n* = 3, 12%). The Low Vision Quality of Life Questionnaire, Clinical Activity Score, and System Usability Score each appeared twice (8%) with the remaining PROs featuring once each. The full list of unique validated PROs is viewable in Supplementary Material.

For the 14 articles with an interventional study design, PROs were used as a primary end point in 5 (35.7%) articles and as a secondary end point in the remaining 9 (64.3%) articles.

**Table 1:** Patient reported outcome characteristics in articles included in the systematic review of patient reported outcomes and ophthalmic artificial intelligence

Most Common PROMs	Number of Articles
National Eye Institute Visual Function Questionnaire EuroQol-5 Dimension Questionnaire (EQ-5D) Graves Ophthalmopathy Quality of Life Questionnaire Low Vision Quality of Life Questionnaire Clinical Activity Score System Usability Score	5 (20%) 3 (12%) 3 (12%) 2 (8%) 2 (8%) 2 (8%)
PROMs by type	
Patient Reported Outcome Measure Patient Reported Experience Measure Social Media Usability Measure	25 (44.2%) 19 (36.5%) 7 (13.5%) 3 (5.8%)
PROMs by specificity	
Generic Disease-specific Both	29 (58%) 21 (40%) 1 (2%)
PRO interaction with AI	
Input Output Both Evaluator metric	22 (44%) 0 (0%) 3 (6%) 25 (50%)
PROM end point position for clinical trials	
Primary Secondary	5 (35.7%) 9 (64.3%)

Key: AI = Artificial Intelligence. PROM = Patient Reported Outcome Measure

### AI characteristics

The articles were also analysed based on the characteristics of the AI algorithm (summarised in Table 2). Within the retrieved articles, AI was most often tasked with screening (*n* = 13, 25.5%) and statistical modelling (*n* = 12, 23.5%). This was followed by monitoring and supportive care (*n* = 8, 15.7%), sentiment analysis (*n* = 7, 13.7%), and diagnosis (*n* = 6, 11.8%). Treatment (*n* = 3. 5.9%) and patient education (*n* = 2, 3.9%) were the least common roles for AI models. Specific outcomes for the included studies are viewable in Supplementary Tables 1–3.

Information regarding the AI models’ intended user was also extracted and overwhelmingly, AI was designed for use by clinicians (*n* = 43, 86%), with only a small number being designed for use by the patient (*n* = 6, 12%), and only a single (2%) model intended for use by both clinicians and patients.

Following on from this, the form of the AI technology was almost exclusively computer software (*n* = 44, 88%) and all AI models designed for clinician use or both clinician and patient use came in the form of computer software. There were very few articles which described other forms of AI technology such as smart device applications (*n* = 3, 6%), wearable technology (*n* = 1, 2%), a video game (*n* = 1, 2%), and a chat bot (*n* = 1, 2%). It was noted that all AI designed for patient use was comprised of these consumer-ready products.

More specifically, of the three smart device applications, one app used AI to convert video from a connected camera into a soundscape to help low-vision patients navigate the environment, another app employed a large-language model AI to answer patient questions and provide education about myopia, and the last app was used to monitor visual acuity at home and flagged patients with likely deteriorations of their diabetic macular oedema for earlier follow up. The wearable took the form of a low-vision headset using AI to synthesise video information gathered from the mounted cameras, the video game as a means for adaptive visual rehabilitation in children with cortical vision loss, and the chat bot facilitated automated post cataract phone reviews and flagged patients requiring additional follow up. These consumer-ready AI technologies employed PROs exclusively as evaluator metrics.

**Table 2:** Artificial intelligence characteristics in articles included in the systematic review of patient reported outcomes and ophthalmic artificial intelligence

Role of AI	Number of Articles
Screening Statistical Modelling Monitoring and Supportive Care Patient Experience or Sentiment Analysis Diagnosis Treatment Patient Education	13 (25.5%) 12 (23.5%) 8 (15.7%) 7 (13.7%) 6 (11.8%) 3 (5.9%) 2 (3.9%)
Intended User	
Clinician Patient Both	43 (86%) 7 (12%) 1 (2%)
Form of AI	
Computer Software Smart Device Application Wearable Technology Video Game Chat Bot	44 (88%) 3 (6%) 1 (2%) 1 (2%) 1 (2%)

Key: AI = Artificial Intelligence

## Discussion

### Summary of results

Emerging evidence highlights the expanding role of PROs and AI in understanding and enhancing ophthalmic quality of life across various ophthalmic conditions. The study selection process found a total of 50 articles meeting the eligibility criteria from which 24 unique validated PROs were identified. The results demonstrated a steady year on year increase in PRO utilisation in AI models from 2020 to 2024 with an all-time high of 16 articles in 2024. Inclusion of PROs was highly concentrated in the most economically advanced countries with countries possessing a GDP over 10 trillion US dollars such as the USA (28%) and China (24%) producing the greatest number of articles. The next closest country, the UK, trailed far behind producing only 6% of included articles. When categorised by ophthalmic subspecialty, PROs appeared most frequently in medical retina (28%), followed by glaucoma (14%), and cornea (14%) related articles.

Patient reported outcomes tended to be generic (58%) rather than disease-specific (40%) and were most often used as evaluator metrics (50%) or as input (44%) for the AI model. For the 14 interventional studies found, PROs were used as a primary end point in 5 (35.7%) articles and as a secondary end point in 9 (64.3%) articles.

Artificial intelligence technologies predominantly took the form of computer software (88%) designed for clinician use (86%). There were few studies applying PROs to consumer ophthalmic AI technology such as smart device applications (6%), wearables (2%), video games (2%), and chat bots (2%). Accordingly, the AI was most often asked to perform screening tasks (25.5%), statistical modelling (23.5%) or to act in a monitoring and supportive role (15.7%).

### Previous reviews

This review provides unique data from a comprehensive systematic review of the literature on the interactions between PROs and ophthalmic AI technology. As previous studies have investigated the role of PROs and AI in medicine as a whole or have explored the utility of PROs within other specialties without addressing AI [53–61].

A review of PROs and AI in medicine by Pearce et al. in 2022, explored the utilisation of PROs in clinical trials that tested an AI health technology. The results of their analysis suggested that while the application of PROs was steadily increasing in the field of medicine as a whole, the inclusion of PROs in clinical trials investigating AI technologies for the eye and adnexa were severely lacking compared to other clinical areas [14]. The decision to focus on validated PROs may have reduced the comprehensiveness of their search, especially given the even split of validated (50%) and non-validated (50%) PROs found in our review. Furthermore, the number of articles incorporating PROs is increasing year on year as demonstrated in Fig. 2. By clinical trials alone, our review found a fivefold increase (*n* = 15) in ophthalmology relevant trials since Pearce et al.’s review (*n* = 3), suggesting that a re-evaluation of the literature was overdue. Nonetheless, the consistent year-on-year increase in studies including PROs and overall small sample size of included articles (*n* = 50) in this review, are in agreeance with Pearce et al.’s findings.

### Challenges to wider adoption and future research

While the steady increase in utilisation of PROs speaks to a growing importance of the patient perspective in ophthalmology, the distribution and characteristics of the PROs and AI found in this review suggest that several barriers exist to fully realising the potential and value of PROs in the development and evaluation of ophthalmic AI.

Despite an increasing trend in the number of articles incorporating PROs each year, the overall number of studies found to date remains small (*n* = 50) implying that research interest in PROs remains low. This is despite the fact that regulatory bodies such as the US Food and Drug Administration (FDA) have recommended PROs to be part of a complete outcome assessment [12]. Furthermore, the concentration of articles in economically advanced countries like the USA (28%) and China (24%) may indicate funding can be allocated to investigate the patient perspective and experience in only the most well-resourced settings. Given the novelty of AI to ophthalmology and medicine as a whole, this is not unexpected as traditional objective outcomes like efficacy, validity and safety of AI tools often need to be demonstrated prior to less well-established subjective measures, like PROs, before they can be considered for clinical implementation [13]. Although not unexpected, the ultimate goal of healthcare and healthcare research is improving quality of life, and thus including PROs is essential for the comprehensive evaluation of any intervention.

Similarly, AI itself is not yet well established in ophthalmology and this is supported by the small number of interventional trials found in this review (*n* = 14), the characteristics of the AI identified, and our previous review on ophthalmic registries and AI [2]. The AI models in this review were most often designed for clinician use (86%), tasked with clinician-relevant tasks like screening (25.5%) and statistical modelling (25.5%), and were seldom in a form designed for consumer use (cumulatively 12%). These characteristics are contrasted against the results of Pearce et al. (2022) who found that from 152 interventional trials employing at least one PRO in all fields of medicine, 95 (62.5%) trials tested AI technologies designed exclusively for patient use, with the most common roles for AI being treatment (*n* = 90, 59.2%) and monitoring or supportive care (*n* = 32, 21.1%), and over 100 (> 65%) trials employed a form of consumer-focused health technology such as smart device applications, wearables, chat bots, video games, and online platforms [14]. These fundamental differences in role of AI, intended end user, and form-factor suggest that compared to other fields in medicine, the integration of AI into ophthalmology is still in the early phases of adoption and exploration where objective outcomes data are paramount and subjective reports of patient health status, experience and product usability are deprioritised [62, 63].

Another issue likely contributing to the underutilisation of PROs in ophthalmic AI, is simply the lack of suitable PRO data to incorporate into AI algorithms. With few exceptions, AI algorithms rely on vast amounts of data to refine their outputs and this data often needs to be curated into a standardised format that is usable by the algorithm [64]. Unlike traditional objective trial outcomes data which has accumulated over the course of decades and has been collated into structured formats within registries or large randomised controlled trials, the accumulation of PROs data has only begun in earnest within the last decade with the current results and other reviews finding that studies incorporating PROs were sporadic prior to 2018 [14]. Beyond the volume of data collected, the organisation and validity of the data is important and the large proportion of articles utilising non-validating PROs (50%) in this review raises concerns that a large volume of the data currently being collected may not be suitable to use as inputs into an AI model. Validated PROs not only benefit from rigorous testing of their contents and psychometric properties to ensure a true measurement of the underlying outcome, but their questions are also standardised and fully transparent which make them ideal training inputs for AI algorithms [65, 66]. Modern psychometric methods such as Rasch analysis and other item response theory-based models, in particular, enable precise and scientifically robust measurement of PROs [67].

In light of these challenges, the utility of PROs remains largely unrealised in ophthalmic AI. While some of these barriers will be addressed over time with the maturing of AI technology within ophthalmology, the increasing focus on providing patient-centred care, and steady accumulation of PROs data, ensuring that this collected data is suitably organised for training AI will require a concerted effort from the scientific community. It is recommended that stakeholders continue to invest into the development and refinement of validated ophthalmic PROs that are applicable to wide variety of research settings and will not unduly increase the burden on researchers and patients to increase uptake and improve utilisation of validated PROs. Furthermore, incorporation of these validated PROs into an ophthalmic registry for routine data collection may significantly accelerate data collection to the critical volume required to actualise AI research within ophthalmology.

### Study limitations

Although the review benefited from a robust and comprehensive search methodology, the restriction of included articles to those available in English resulted in the exclusion of 5 potentially viable articles [68–72]. Given the relatively small number of included articles (*n* = 50), the exclusion of these articles may be a source of bias that impacts the interpretation of the results.

Furthermore, despite the reduction in publication bias and increased breadth of articles retrieved by the search strategy as a result of including conference abstracts and clinical trials, the brevity and lack of standardised reporting items for these article types raised issues during data synthesis. Some articles would refer to the PRO as simply a “patient satisfaction questionnaire” and other articles would refer to their AI tool as “artificial intelligence” with no further information or explanation thus precluding a more detailed synthesis of the data. In preliminary searches, the heterogeneity of reporting standards between articles made it clear that the inclusion of more detailed data extraction columns such as ‘type of algorithm’ or ‘PRO delivery format’ would lead to issues with missing data and an increased number of excluded articles. The development of standardised PRO reporting items is essential to allow for high quality data synthesis in future reviews.

## Conclusion

In this systematic review of the literature, PROs were found to be underutilised in studies investigating ophthalmic AI technology. Several challenges to realising the full potential of PROs were identified including low research priority, the nascent state of AI in ophthalmology, and high-quality PROs data not yet reaching critical mass for full scale incorporation into AI algorithms. The results of this review encourage stakeholders and future researchers to invest in the development of robust validated PROs and to incorporate these PROs into routine data collection to enable the development of ophthalmic AI that not only addresses objective outcomes, but also provides care best aligned with expressed wishes and makes the greatest meaningful difference for the patient.

## Supplementary Information

Below is the link to the electronic supplementary material.

Supplementary Material 1

## Data Availability

All data and material are available upon reasonable request.

## Abbreviations

AI: Artificial intelligence
FDA: Food and Drug Administration
GDP: Gross domestic product
PREM: Patient reported experience measure
PRO: Patient reported outcomes
PROM: Patient reported outcome measures
