# Supplementary material for: Artificial intelligence and patient reported outcomes in ophthalmology
Source: J Patient Rep Outcomes. 2026 Mar 7;10:60. doi: 10.1186/s41687-026-01033-0 (PMC13079251; doi:10.1186/s41687-026-01033-0)
Supplement: Supplementary file 1 — Supplementary Material 1 [file 41687_2026_1033_MOESM1_ESM.docx]

**Supplementary Table 1.** Summary of evidence for journal articles included in the systematic review applying PROMs as an evaluator metric for AI

| **Publication Details** | **Aim of Study** | **Role of AI** | **PROM Name** | **PROM Form and Purpose** | **PROM Relevant Findings** | **Conclusions** |
| --- | --- | --- | --- | --- | --- | --- |
| Feasibility study of diabetic retinopathy detection in type II diabetic patients based on explainable AI  Lalithadevi et al. (2023) | To evaluate an AI model predicting risk of DR among study participants | Diagnosis | Unnamed | Questionnaire: to assess patient perception of AI reliability, comfort level during screening, willingness to participate in the future, overall perspective, and satisfaction with DR screening | Participant responses: n = 172;  50% felt AI predictions were reliable;  61.62% felt comfortable during AI screening;  66.86% willing to participate in future screenings if needed;  62.21% overall satisfied with AI screening;  Between 6.39-11.63% of participants did not provide a comment on these questions | Patients found AI DR screening to be reliable, comfortable, would be willing to participate again, and were satisfied with the overall experience |
| Fully automatic postoperative appearance prediction system for blepharoptosis surgery with image-based deep learning  Sun et al. (2022) | To predict post blepharoplasty appearance using AI and evaluate the generated images objectively and subjectively | Monitoring and supportive care | Unnamed | Survey: To assess patient and ophthalmologist satisfaction with predicted post op outcome, patient extent of anxiety or hesitation due to unknown postoperative appearance, and extent of relief due to the prediction | Participant responses: n = 6  Ophthalmologists: n = 4  Postoperative prediction vs. end result paired photos of eyes: n = 750.  Patients and ophthalmologists were ‘highly satisfied’ with 56% of paired photos;  ‘satisfied’ with 35.7%;  ‘neutral’ with 8%;  ‘not satisfied’ with 0.3%;  100% of patients were anxious or hesitant due to the unknown post-op outcome;  4 patients were ‘highly relieved’ and 2 were ‘relieved’ due to the predictions | High patient satisfaction with the AI’s postoperative appearance predictions |
| Augmentation of telemedicine post-operative follow up after oculofacial plastic surgery with a self-guided patient tool  Ashraf et al. (2023) | To evaluate a web-based tool designed to augment telemedicine post-op visits after periocular surgery | Statistical modelling | Unnamed | Survey: Five-point Likert scale and perceived time using tool to assess patient satisfaction with telemedicine follow up tool; higher score is better for Likert scale questions; lower score is better for minutes using tool | Participant responses: n = 28;  Overall telemedicine experience: 4.6 ± 0.7;  Comfort with telemedicine visit: 4.6 ± 0.9;  Ease of using tool: 3.6 ± 1.1;  Usefulness of tool: 4.7 ± 0.6;  Time spent on pre-visit tool: 15.3 ± 9.0;  Patient reported duration of telemedicine visit: 30.1 ± 17.7;  Patient reported duration of typical clinic visit: 150.6 ± 118.2; | Augmented telemedicine follow-up after oculoplastic surgery was associated with high patient satisfaction, rare conversion to clinic evaluation, and few related post-op complications |
| Autonomous artificial intelligence increases screening and follow-up for diabetic retinopathy in youth: the ACCESS randomized trial  Wolf et al. (2023) | To evaluate the effect of a non-mydriatic AI DR screening tool on exam completion rates in a racially and ethnically diverse cohort of youths with T1 and T2DM as compared to a standard dilated DR screening examination | Screening | Unnamed | Survey: To assess satisfaction with and acceptability of the intervention | Participants who answered the survey in the AI arm: n = 80;  Patients who answered the survey in the control arm: n = 15;  Patients satisfied:  Time to complete DR screening: AI = 92.5% vs. control = 100%;  Time to receive results: AI = 95% vs. control = 93.3%;  Overall experience: AI = 96.2% vs. control 93.3%;  Patients who would choose:  POC AI DR screening next time: AI = 84.8% vs. control 93.3%;  Standard dilated DR screening with an eye care provider next time: AI = 57% vs. control = 80% | Patients were satisfied with the experience of AI DR screening and a significant proportion would choose the non-mydriatic AI DR screening over the dilated eye care provider DR screening |
| Clinical efficacy of a head-mounted device for central vision loss  Gu et al. (2024) | Evaluate the clinical efficacy of a novel AI-integrated head-mounted device for patients with CVL | Monitoring and supportive care | LVQoL Questionnaire (25 item) | Questionnaire: To assess the effect of the AI headset on VRQoL; higher score is better | Participant responses: n = 41;  Baseline VRQoL score without headset: 52.05 ± 3.75;  VRQoL with headset: 60.95 ± 3.80;  Percentage of improvement: 17.10% (p <0.001);  Patients with a statistically significant improvement in VRQoL score: 68.29% | AI head-mounted headset was clinically effective in improving VRQoL in patients with CVL |
| Diagnostic efficacy and therapeutic decision-making capacity of an artificial intelligence platform for childhood cataracts in eye clinics  Lin et al. (2019) | To compare the diagnostic efficacy and treatment decision-making capacity for childhood cataract between an AI model and ophthalmologists in real-world clinical settings | Diagnosis and treatment | Unnamed | Questionnaire: Five-point Likert scale to assess patient and guardian satisfaction with diagnostic accuracy and efficiency | Patients: n = 345;  Overall satisfaction for the AI model: 3.47 ± 0.501;  Overall satisfaction for senior consultants: 3.38 ± 0.554;  p = 0.007 | AI model exhibited less accuracy compared with senior human consultants in diagnosing childhood cataracts and making treatment decisions, but has the capacity to assist doctors in clinical practice in its current state |
| Feasibility and acceptance of artificial intelligence-based diabetic retinopathy screening in Rwanda  Whitestone et al. (2023) | To evaluate feasibility and patient acceptance of AI-based DR screening in Rwandan diabetes clinics | Screening | Unnamed | Survey: To assess patient satisfaction with screening process | Participant responses: n = 827;  Patients reporting ‘high’ satisfaction with screening process: n = 823 (99.5%);  Patient preference for AI versus human expert: n = 527 (63.7%) | AI DR-screening led to accurate referrals from diabetes clinics in Rwanda and high rates of patient satisfaction |
| Feasibility and patient experience of a pilot artificial intelligence-based diabetic retinopathy screening program in Northern Ontario  Bhambhwani et al. (2024) | To assess the feasibility, implementation, and patient experience of an AI DR detection tool in a primary care setting in Ontario | Screening | Unnamed | Questionnaire: Five-point Likert scale to assess patient satisfaction and overall experience with the process; higher score is better | Participant responses: n = 184;  Mean satisfaction for:  Eye examination: 4.8 ± 0.64;  Time to receive results from human: 4.7 ± 0.7;  Time to receive results from AI: 4.8 ± 0.7;  Overall satisfaction: 4.8 ± 0.6;  Value to patients for:  Not having to wait for a separate visit for an eye exam: 4.8 ± 0.5;  Not having to use dilating drops: 4.9 ± 0.5;  Patient preference for using an AI system rather than a physician: 4.7 ± 0.7 | Screening for DR using AI in a primary care setting is feasible and acceptable |
| Robotic process automation support in telemedicine: Glaucoma screening usage case  Thainimit et al. (2022) | To evaluate the time-effectiveness and feasibility of integrating robotic process automation and machine learning in glaucoma tele-screening via a mobile application | Screening | Unnamed | Questionnaire: Ten-point scale to assess patient satisfaction with the mobile application; higher score is better | Participant responses: n = 68;  Mean satisfaction with:  App layout: 8.35 ± 1.13;  Content coherence: 8.23 ± 1.03;  Content completeness: 7.9 ± 1.07 | Integration of robotic process automation and machine learning allows for efficient resource management and may allow for high volume ocular screening |
| AI = artificial intelligence. CVL = cortical vision loss. DR = diabetic retinopathy. LVQoL = low vision quality of life. POC = point of care. PROM = patient reported outcome measure. T2DM = type 2 diabetes mellitus. VRQoL = vision related quality of life. | | | | | | |

**Supplementary Table 2.** Summary of evidence for journal articles included in the systematic review applying PROMs as an input for AI

| **Publication Details** | **Aim of Study** | **Role of AI** | **PROM Name** | **PROM Form and Purpose** | **PROM Relevant Findings** | **Conclusions** |
| --- | --- | --- | --- | --- | --- | --- |
| A multifactorial screening model based on the Graves Ophthalmopathy Quality of Life Scores in dysthyroid optic neuropathy  Liang et al. (2024) | To assess the GO-QOL’s ability to screen for DON and to build and investigate the efficacy of a screening model that uses GO-QOL to detect DON | Screening | GO-QOL | Questionnaire: A sixteen-item questionnaire to assess visual functioning QoL and changed appearance QoL in patients with GO; higher score is better | Participant responses: n = 194;  DON group median for:  Visual function score: 33.18 ± 24.52;  Appearance score: 60.08 ± 24.82;  Non-DON group median for:  Visual function score: 81.26 ± 17.39;  Appearance score: 76.14 ± 27.56 | GO-QOL can be an efficient tool for screening for DON especially with regards to visual function scores. A visual function cutoff score of 58 was beneficial for the purposes of clinical follow-up, diagnosis, and treatment in DON. |
| Application of neural network model in assisting device fitting for low vision patients  Dai et al. (2020) | To evaluate the efficacy of an AI model in fitting low-vision assistive devices for visually impaired participants in Fujian | Monitoring and supportive care | LVQoL Questionnaire (25 item) | Questionnaire: To assess QoL in patients with low vision; higher score is better | Participant responses: n = 629;  Quality of life scores were inversely correlated with level of visual impairment | AI model taking visual function, rehabilitation needs, and quality of life scores as input achieved high accuracy in low-vision aid device fitting |
| Application of sentiment and word frequency analysis of physician review sites to evaluate refractive surgery care  Vought et al. (2024) | To assess patient satisfaction with their ophthalmologist and understand aspects of care that are valued by patients | Sentiment analysis | Not applicable | Social media and forum posts: Written and star reviews of ophthalmologists on Healthgrades; Sentiment analysis was performed on written reviews to generate a compound score; higher score is better | Data collected from: 254 specialists and 3104 reviews;  Overall satisfaction for all ophthalmologists: 4.38 stars / 0.69 compound score;  No statistically significant difference was noted in average star rating or compound score when scores were stratified by gender or location;  Physicians with fewer years of practice had higher ratings than peers with greater years in practice (4.56 vs. 4.20 stars; 0.71 vs. 0.67 compound score);  The most positive reviews often included words such as: ‘surgery’, ‘staff’, ‘procedure’, ‘experience’, ‘professional’;  Negative reviews included words such as: ‘surgery’, ‘office’, ‘staff’, ‘time’, ‘insurance’ | Overall high patient satisfaction with ophthalmologists and often non-clinical factors such as interactions with office staff, insurance coverage, wait times and physician bedside manner impacted patient reviews |
| Association of lesion location and functional parameters with vision-related quality of life in geographic atrophy secondary to AMD  Kunzel et al. (2024) | To determine how structural and functional parameters influence the VRQoL in patients suffering from GA secondary to AMD | Statistical modelling | NEI-VFQ 25 | Questionnaire: To assess various aspects of vision-related functioning; higher score is better | Participant responses: n = 82;  Mean VRQoL:  Composite score: 70;  Near activities score: 50;  Distance activities score: 58;  VRQoL for distance and near activities was most strongly correlated with the ETDRS inner lower and inner left subfields of the better eye respectively;  For patients with foveal-sparing GA, low luminance VA of the better eye was the most influential variable across all VRQoL scales | The pivotal role of GA location in relation to VRQoL in GA patients was demonstrated, such findings are critical in informing treatment decisions and refining the planning of interventional trials |
| Evaluating patient experiences in dry eye disease through social media listening research  Cook et al. (2019) | To use social media listening to understand patient experiences and unmet needs in DED | Sentiment analysis | Not applicable | Social media and forum posts: Relevant posts on various open-access social media platforms (Twitter, blogs, news, and forums) detailing patient experiences with DED | Unique patient posts found by AI aggregator tool: n = 1192;  Key themes of discussion: management (1393), symptoms (901), causes (409), diagnosis (137), associated comorbidities (187);  Most common symptoms mentioned: eye dryness, pain, blurry vision;  Most often mentioned activities negatively affected by DED: workplace difficulties, commute/driving, use of electronic devices, reading, inability to wear contact lenses;  Key unmet needs: lack of awareness of DED, insufficient access to essential information about the disease, lack of treatment providing lasting relief | Insights gathered from social media listening strengthen our understanding about patient experiences and their unmet needs in DED which may inform early drug development processes, market access strategies and stakeholder discussions |
| Exploring disease perception in Behcet's syndrome: combining quantitative and a qualitative study based on a narrative medicine approach  Marinello et al. (2023) | To explore disease experience and perceptions in BS patients | Statistical analysis | Unnamed | Questionnaire: Five-point Likert scale to assess the impact of disease of different aspects of patients’ lives (work, family, social relations, etc.) | Participant responses to survey: n = 207;  Stories collected: n = 43;  Patients generally reported concerns regarding the impact of BS on their life and families;  BS is perceived to significantly affect patients’ perception of themselves and the world around them, especially in terms of working life and personal relationships;  Participants were clustered by AI into three distinct groups based on hope for treatment, acceptance of disease, and contact with other BS patients;  Acceptance of the disease had a strong impact on the way patients perceived themselves and their hope for the future | This study is the first to address patient disease perception using a narrative medicine approach which may encourage the scientific community to improve the journey for BS patients |
| Extended screen time and dry eye in youth  Muntz et al. (2021) | To evaluate spontaneous blink rates, dry eye symptomatology and screen use habits of young extended screen time users | Statistical modelling | DEQ-5, SANDE | Questionnaire: To assess dry eye symptomatology and impact of QoL; lower scores are better | Participant responses: n = 456;  Mean DEQ-5 score (IQR): 11 (8-13);  Mean SANDE score: 33 ± 18;  Mean impact on:  Daily life tasks: 25 ± 21;  VRQoL: 28 ± 22 | Extended screen time in a young population was associated with blinking behaviours and symptomology consisted with DED; Routine clinical screening, education, and developing official guidance on safe screen use may prevent an accelerated degradation of ocular surface health and QoL in young people |
| Facial expression of patients with Graves' orbitopathy  Lei et al. (2023) | To analyse the facial expressions of GO patients and explore links to clinical signs and QoL | Statistical modelling | GO-QOL, CAS | GO-QOL: A sixteen-item questionnaire to assess visual functioning QoL and changed appearance QoL in patients with GO; higher score is better  CAS: A clinical tool used to evaluate disease activity with two questions assessing patient reported spontaneous retrobulbar pain and pain on attempted up or down gaze; lower score is better | Participant responses (GO-QOL): n = 126;  Participant responses (CAS): n = 943);  Mean QoL:  Visual function score: 60.3 ± 24.0;  Appearance score: 50.5 ± 27.0;  Number of participants reporting: Spontaneous retrobulbar pain: n = 386 (40.9%);  Pain on attempted up or downgaze: n = 161 (17.1%) | AI model was able to accurately characterise facial expressions of GO patients; Facial expressions are a relevant factor in the severity, activity, and QoL in GO |
| Identification of noncompliant glaucoma patients using Bayesian networks and the Eye-Drop Satisfaction Questionnaire  Nordmann et al. (2010) | To identify poorly compliant glaucoma patients using Bayesian networks and EDSQ | Monitoring and supportive care | EDSQ | Questionnaire: A 21-item questionnaire to address six dimensions of attitudes to eye-drop treatment (treatment concern, disease concern, satisfaction with the patient-physician relationship, positive beliefs, and self-declared compliance); lower score is better for treatment concerns and disease concern; higher score is better for all other dimensions | Participant responses: n = 169;  Low compliance was associated with two combinations of factors:   1. Age < 77.5 years, self-declared compliance score <89.5, and a patient-clinician relationship score <67.5 2. Age >77.5 years, self-declared compliance score >89.5, and a patient-clinician relationship score <67.5   High compliance was associated with: age <77.5 years, self-declared compliance score >89.5, and patient-clinician relationship score >67.5 | Central role of the patient-physician relationship in compliance to glaucoma treatment; Age, self-declared compliance, and patient satisfaction with the patient-physician relationship are factors worth exploring before considering changes in glaucoma management |
| Lower visual acuity predicts worse utility values among patients with type 2 diabetes  Smith et al. (2008) | To estimate quality of life impact of vision loss in T2DM using NLP and regression modelling | Statistical modelling | EQ-D5 | Questionnaire: Five-item questionnaire designed to evaluate QoL in five key areas (mobility, self-care, usual activities, pain/discomfort, and anxiety/depression); scores were converted to utility values with 1.0 corresponding to perfect health | Participant responses: n = 2074;  Utility value for the healthiest cohort of patients (n = 54, 20/20 vision, duration of diabetes <5 years, no insulin use, no CVD, no neuropathic symptoms): 0.94 ± 0.08;  Utility value with VA 20/40: 0.91 ± 0.02;  Utility value with VA =< 20/80: 0.85 ± 0.04 | Lower visual acuity correlates with lower EQ-5D utility values in a representative population of patients with T2DM |
| Patient experience of Sjogren's disease and its multifaceted impact on patients' lives  Perella et al. (2023) | To explore patient-reported impact of Sjogren's disease using social media listening. | Sentiment analysis | Not applicable | Social media and forum posts: Relevant posts on various open-access social media platforms (Twitter, Reddit, and forums) detailing patient experiences with Sjogren’s | Unique patient posts found by AI aggregator tool: n = 4231;  Most common symptoms domains:   1. Pain (51%) 2. Dry mouth and throat (41%) 3. Fatigue, energy and sleep (40%) 4. Emotional balance (33%) 5. Dry eye (32%)   Most impactful symptom domains:   1. Emotional balance 2. Fatigue, energy, and sleep 3. Pain 4. Dry eye 5. Dry mouth and throat   Impacted HRQoL domains by commonness:   1. Daily functioning (72%) 2. Social wellbeing (35%) 3. Financial health (31%) 4. Psychological wellbeing (30%) | Sjogren’s affects diverse aspects of patients’ lives, with symptoms beyond dry eyes and mouth and impacts to daily living and functioning; Given that symptomatology is unique to each patient, it is important to measure impacts on HRQoL to assess patient outcomes and management options in routine clinical practice |
| Patient perceptions of disease burden and treatment of myasthenia gravis based on sentiment analysis of digital conversations  Anderson et al. (2024) | To explore unprovoked patient-reported perspectives on living with MG through social media | Sentiment analysis | Not applicable | Social media and forum posts: Relevant posts on various open-access social media platforms (forums, Reddit, topical sites, Facebook, Instagram, Twitter, TikTok, Youtube, blogs, and comments) detailing patient experiences with MG | Unique digital conversations found by AI aggregator tool: n = 13234;  Most common topics of discussion:   1. Diagnosis (29%) 2. Living with MG (28%) 3. Symptoms (24%) 4. Treatment (19%)   Most common symptoms described:   1. Ocular problems (21%) 2. Facial muscle problems (18%) 3. Fatigue (18%)   Sentiment of conversation:   1. Positive (2%) 2. Negative (59%)   Most common themes of negative conversations:   1. Impact on life (29%) 2. Misdiagnosis (27%) 3. Treatment issues (24%) 4. Symptom severity (20%)   Most common treatment issues:   1. Side effects (36%) 2. Lack of efficacy (33%) 3. Misdiagnosis (21%) 4. Cost/insurance (10%) | High degree of concern among patients with MG regarding burden of disease, misdiagnosis, treatment and side effects, highlighting the limitations of currently available MG treatments and the need for improved symptom control options with more manageable side effects |
| Physician review websites: understanding patient satisfaction with ophthalmologists using natural language processing  Jo et al. (2023) | To assess patient satisfaction with ophthalmologists through applying NLP to ratings and reviews on physician review websites | Sentiment analysis | Not applicable | Social media and forum posts: Reviews and star ratings for ophthalmologists on Healthgrades.com | Unique web reviews found: n = 16700;  Average star ratings for male vs. female ophthalmologists: 4.61 vs. 4.55, p < 0.001;  Average sentiment analysis score for male vs. female ophthalmologists: 0.645 vs. 0.624, p < 0.002;  Average star ratings by ophthalmologist years of age (<40, 40-49, 50-59, >60): 4.72 vs. 4.51 vs. 4.38 vs. 4.40, p < 0.001;  Average sentiment analysis score by ophthalmologist years of age (as above): 0.691 vs. 0.616 vs. 0.586 vs. 0.590, p < 0.001;  Words most commonly associated with positive reviews: ‘friendly’ ‘caring’, ‘kind’, ‘comfortable’;  Words most commonly associated with negative reviews: ‘rude’, ‘unprofessional’, ‘arrogant’, ‘condescending’, ‘waiting’, ‘rushed’ | Younger and male ophthalmologists received higher star ratings and sentiment analysis scores; Pleasant personality and visit effectiveness were most commonly associated with positive reviews, whereas wait times or unpleasant personality were most likely to denote a negative review |
| VRQoL after unilateral occipital stroke  Dogra et al. (2024) | To determine if VRQoL is impacted by time since stroke | Statistical modelling | NEI-VFQ 25, Neuro10 | NEI-VFQ: To assess various aspects of vision-related functioning; higher score is better  Neuro10: 10-item add neuro-ophthalmic supplement for the NEI-VFQ; higher score is better | Participant responses: n = 95;  Mean NEI-VFQ score for cortical blindness group = 68.2 ± 15.3 vs. control = 93.1 ± 6.8;  Mean Neuro10 score for cortical blindness group = 73.0 ± 15.8 vs. control = 95.0 ± 5.0;  Simple linear regressions demonstrated a significant correlation between NEI-VFQ (p = 0.0046) and Neuro10 (p = 0.0039) scores and time post stroke | VRQoL improves with time post occipital stroke regardless of visual deficit size or patient age at insult; This may reflect natural development of compensatory strategies and lifestyle adjustments |
| AI = artificial intelligence. AMD = age related macular degeneration. BS = Behcet's syndrome. CAS = clinical activity score. CVD = cardiovascular disease. DED = dry eye disease. DEQ-5 = dry eye questionnaire 5. DON = dysthyroid optic neuropathy. DR = diabetic retinopathy. EDSQ = eye drop satisfaction questionnaire. EQ-5D = EuroQoL 5-dimension. ETDRS = Early Treatment of Diabetic Retinopathy Study. GA = geographic atrophy. GO = Graves Ophthalmopathy. GO-QOL = Graves Ophthalmopathy Quality of Life. HRQoL = health-related quality of life. IQR = interquartile range. LVQoL = low vision quality of life. MG = myasthenia gravis. ML = machine learning. NEI-VFQ 25 = National Eye Institute Visual Functioning Questionnaire 25. Neuro10 = neuro-ophthalmic supplement 10. NLP = natural language processing. PROM = patient reported outcome measure. QoL = quality of life. SANDE = symptom assessment in dry eye. T2DM = type 2 diabetes mellitus. VA = visual acuity. VRQoL = vision related quality of life. | | | | | | |

**Supplementary Table 3.** Summary of evidence for journal articles included in the systematic review applying PROMs as both Input and Output for AI

| **Publication Details** | **Aim of Study** | **Role of AI** | **PROM Name** | **PROM Form and Purpose** | **PROM Relevant Findings** | **Conclusions** |
| --- | --- | --- | --- | --- | --- | --- |
| Ensemble deep learning diagnostic system for determining Clinical Activity Scores in thyroid-associated ophthalmopathy  Yan et al. (2024) | To develop and evaluate a multimodal deep learning system to predict CAS in TED | Screening | CAS | Clinical tool: To evaluate disease activity with two questions assessing patient reported spontaneous retrobulbar pain and pain on attempted up or down gaze; lower score is better | Participant responses: n = 156;  Average CAS: 2.2;  Specific data on spontaneous retrobulbar pain and pain on attempted up/down gaze was not published despite being incorporated into training the deep learning algorithm | Deep learning system developed in this study more accurately assessed the clinical activity of TED than traditional methods relying solely on facial images |
| Evaluation of various machine learning methods to predict vision-related quality of life from visual field data and visual acuity in patients with glaucoma  Hirasawa et al. (2014) | To assess whether ML algorithms can accurately predict VRQoL from VF and VA in glaucoma patients | Monitoring and supportive care | Sumi Questionnaire | Questionnaire: A 30-item tool assessing seven tasks: legibility of letters, legibility of sentences, walking, using public transportation, dining, dressing, miscellaneous activities; lower score is better | Participant responses: n = 164;  RMSE range for ML algorithms (RF, SVM, Boost) predicting VRQoL: 1.99 – 2.21;  RMSW range for linear models predicting VRQoL: 2.35 – 3.15;  Actual VRQoL scores collected were not reported | Machine learning methods, especially RF methods, can be used to estimate VRQoL in the clinical setting, to help clinicians better predict VRQoL based on standard clinical measurements |
| Investigating factors influencing quality of life in thyroid eye disease  Zhang et al. (2025) | To identify factors affecting QoL in TED using AI | Statistical modelling | GO-QOL, CAS | GO-QOL: A sixteen item questionnaire to assess visual functioning QoL and changed appearance QoL in patients with GO; higher score is better  CAS: A clinical tool used to evaluate disease activity with two questions assessing patient reported spontaneous retrobulbar pain and pain on attempted up or down gaze; lower score is better | Participant responses: n = 211;  Median QoL VF score: 62.29 (95% CI 42.86-93.75);  Median QoL AP score: 62.5 (95% CI 43.75-87.50);  Participants reporting spontaneous retrobulbar pain: n = 57 (27%);  Participants reporting ocular motility pain: n = 37 (17.5%);  Univariate analysis:  Female vs. male QOL VF: 66.76 ± 31.65 vs. 52.16 ± 33.65, p = 0.002;  Female vs male QOL AP: 57.74 ± 29.25 vs. 69.18 ± 27.15, p = 0.006;  QOL-VF was positively correlated with years of education (p = 0.008) and disease duration (p = 0.002);  QOL-VF was negatively correlated with age (p < 0.001), previous IV steroid use (p < 0.001) or other treatments (p = 0.007);  QOL-AP was not significantly correlated with disease severity (p = 0.313) | TED significantly affects patient QoL as demonstrated by ML analysis |
| AI = artificial intelligence. AP = physical appearance. CAS = clinical activity score. GO = Graves Ophthalmopathy. GO-QOL = Graves Ophthalmology quality of life. IV = intravenous. ML = machine learning. PROM = patient reported outcome measure. QoL = quality of life. RF = random forest. RMSE = root mean squared error. SVM = support vector machine. TED = thyroid eye disease. VA = visual acuity. VF = visual field. VRQoL = vision related quality of life. | | | | | | |

**Supplementary Material – Search Strategy**

Search syntaxes for PubMed, Medline, EMBASE, and ClinicalTrials.gov databases

**Concepts:**

1. Population: AI health technologies within ophthalmology
2. Intervention: Patient reported outcome measures

**Search conducted on:** 9/2/2025

**Master key word search for Medline and EMBASE databases:**

(ophthal* OR eye OR vision OR ocular OR glaucoma OR uveitis OR cataract OR cornea OR retina) AND (“artificial intelligence” OR “deep?learning” OR “machine?learning” OR “neural network” OR “natural language processing” OR “prediction model”) AND (“patient reported outcomes” OR PROM OR “quality of life” OR QOL OR “symptom burden” OR “functional status” OR “health behaviors” OR “patient experience” OR “patient satisfaction” OR “patient participation” OR “patient activation”)

**Medline vis OvidSP (1946 – present): n = 89**

| **#** | **Searches** | **Result** |
| --- | --- | --- |
| 1 | exp Ophthalmology/ or ophthal*.mp. | 191710 |
| 2 | exp Posterior Eye Segment/ or eye.mp. or exp Eye Diseases, Hereditary/ or exp Eye Diseases/ or exp Eye/ or exp Eye Injuries/ or exp Anterior Eye Segment/ or exp Eye Infections/ | 1023865 |
| 3 | (vision or ocular).mp. or exp Vision, Ocular/ | 416868 |
| 4 | glaucoma.mp. or exp Glaucoma/ | 87020 |
| 5 | exp Uveitis/ or uveitis.mp. | 44584 |
| 6 | exp Cataract/ or cataract.mp. | 77614 |
| 7 | exp Cornea/ or cornea.mp. | 89750 |
| 8 | retina.mp. or exp Retina/ | 197526 |
| 9 | 1 or 2 or 3 or 4 or 5 or 6 or 7 or 8 | 1234806 |
| 10 | artificial intelligence.mp. or exp Artificial Intelligence/ | 255446 |
| 11 | exp Deep Learning/ or "deep?learning".mp. | 25972 |
| 12 | exp Machine Learning/ or "machine?learning".mp. | 84682 |
| 13 | exp Neural Networks, Computer/ or "neural network".mp. | 127678 |
| 14 | exp Natural Language Processing/ | 7613 |
| 15 | 10 or 11 or 12 or 13 or 14 | 298563 |
| 16 | PROM.mp. or exp Patient Reported Outcome Measures/ | 22150 |
| 17 | QOL.mp. or exp "Quality of Life"/ | 317595 |
| 18 | exp Symptom Burden/ | 137 |
| 19 | exp Functional Status/ | 1745 |
| 20 | exp Health Behavior/ | 379252 |
| 21 | exp Patient Satisfaction/ | 104700 |
| 22 | exp patient participation/ | 30771 |
| 23 | 16 or 17 or 18 or 19 or 20 or 21 or 22 | 688008 |
| 24 | 9 and 15 and 23 | 89 |

**EMBASE via OvidSP (1947 to present): n = 745 articles**

| **#** | **Searches** | **Result** |
| --- | --- | --- |
| 1 | exp Ophthalmology/ or ophthal*.mp. | 284663 |
| 2 | exp Posterior Eye Segment/ or eye.mp. or exp Eye Diseases, Hereditary/ or exp Eye Diseases/ or exp Eye/ or exp Eye Injuries/ or exp Anterior Eye Segment/ or exp Eye Infections/ | 1795076 |
| 3 | (vision or ocular).mp. or exp Vision, Ocular/ | 716141 |
| 4 | glaucoma.mp. or exp Glaucoma/ | 138347 |
| 5 | exp Uveitis/ or uveitis.mp. | 85075 |
| 6 | exp Cataract/ or cataract.mp. | 128447 |
| 7 | exp Cornea/ or cornea.mp. | 154392 |
| 8 | retina.mp. or exp Retina/ | 344913 |
| 9 | 1 or 2 or 3 or 4 or 5 or 6 or 7 or 8 | 2081824 |
| 10 | artificial intelligence.mp. or exp Artificial Intelligence/ | 138510 |
| 11 | exp Deep Learning/ or "deep?learning".mp. | 71464 |
| 12 | exp Machine Learning/ or "machine?learning".mp. | 543395 |
| 13 | exp Neural Networks, Computer/ or "neural network".mp. | 161873 |
| 14 | exp Natural Language Processing/ | 14832 |
| 15 | 10 or 11 or 12 or 13 or 14 | 633191 |
| 16 | PROM.mp. or exp Patient Reported Outcome Measures/ | 76682 |
| 17 | QOL.mp. or exp "Quality of Life"/ | 748171 |
| 18 | exp Symptom Burden/ | 1646 |
| 19 | exp Functional Status/ | 80910 |
| 20 | exp Health Behavior/ | 534619 |
| 21 | exp Patient Satisfaction/ | 183253 |
| 22 | exp patient participation/ | 38592 |
| 23 | 16 or 17 or 18 or 19 or 20 or 21 or 22 | 1545510 |
| 24 | 9 and 15 and 23 | 745 |

**PubMed: n = 193 articles**

(“ophthal*"[Title/Abstract] OR "Ophthalmology"[Mesh] OR “Eye”[MeSH] OR "Vision, Ocular"[Mesh] OR “vision”[Title/Abstract] OR “ocular”[Title/Abstract] OR “glaucoma”[Title/Abstract] OR "Glaucoma"[Mesh] OR “uveitis”[Title/Abstract] OR "Uveitis"[Mesh] OR “cataract”[Title/Abstract] OR "Cataract"[Mesh] OR “cornea”[Title/Abstract] OR "Cornea"[Mesh] OR “retina”[Title/Abstract] OR "Retina"[Mesh]) AND (“artificial intelligence”[Title/Abstract] OR "Artificial Intelligence"[Mesh] OR “deep?learning”[Title/Abstract] OR "Deep Learning"[Mesh] OR “machine?learning”[Title/Abstract] OR "Machine Learning"[Mesh] OR “neural network”[Title/Abstract] OR "Neural Networks, Computer"[Mesh] OR “natural language processing”[Title/Abstract] OR "Natural Language Processing"[Mesh] OR “prediction model”[Title/Abstract]) AND (“patient reported outcomes”[Title/Abstract] OR "Patient Reported Outcome Measures"[Mesh] OR PROM[Title/Abstract] OR “quality of life”[Title/Abstract] OR "Quality of Life"[Mesh] OR QOL[Title/Abstract] OR “symptom burden”[Title/Abstract] OR "Symptom Burden"[Mesh] OR “functional status”[Title/Abstract] OR "Functional Status"[Mesh] OR “health behaviors”[Title/Abstract] OR "Health Behavior"[Mesh] OR “patient experience”[Title/Abstract] OR “patient satisfaction”[Title/Abstract] OR "Patient Satisfaction"[Mesh] OR “patient participation”[Title/Abstract] OR "Patient Participation"[Mesh] OR “patient activation”[Title/Abstract])

**ClinicalTrials.gov: n = 115 trials**

(Condition = Eye Diseases, Intervention: Artificial Intelligence)
